# EGFR-AS1 Promotes Nonsmall Cell Lung Cancer (NSCLC) Progression via Downregulating the miR-524-5p/DRAM1 Axis and Inhibiting Autophagic Lysosomal Degradation

**DOI:** 10.1155/2022/4402536

**Published:** 2022-02-16

**Authors:** Yang Xue, Jing Zhang, Jiguang Hou, Xin Wang

**Affiliations:** ^1^Department of Cardio-Thoracic Surgery, People's Hospital of Deyang City, Deyang, Sichuan 618000, China; ^2^Department of Pulmonary and Critical Care Medicine, Shandong Public Health Clinical Center, Jinan, Shangdong 250013, China; ^3^Department of Radiotherapy, The Second Hospital of Jilin University, Jilin, Changchun 130000, China; ^4^Department of Respiration, Jinan Central Hospital, Cheeloo College of Medicine, Shandong University, No. 105, Jiefang Road, Lixia District, Jinan, Shandong Province 250013, China

## Abstract

Nonsmall cell lung cancer (NSCLC) accounts for the majority of lung cancers. Studies have revealed the regulatory role of lncRNAs in cancer pathogenesis and their potential use as diagnostic and prognostic biomarkers. The epidermal growth factor receptor antisense RNA 1 (EGFR-AS1) has been reported to be upregulated in NSCLC tissues, while its detailed mechanism in lung cancer needs to be explored. DNA damage-regulated autophagy modulator 1 (DRAM1) has been known to act as a tumor suppressor in NSCLC, and miR-524-5p has been reported to be a biomarker in idiopathic pulmonary fibrosis and different lung disorders. Our investigation revealed that EGFR-AS1 is highly expressed in lung cancer tissues, and its knockdown inhibited lung cancer cell invasion and viability and reduced tumor growth *in vivo*. We also found that EGFR-AS1 targets miR-524-5p, and there was a negative correlation between their expressions in lung cancer tissues. Simultaneously, miR-524-5p has been found to promote DRAM1 expression. In addition, the inhibition of miR-524-5p diminished DRAM1 protein expression and promoted lung cancer cell invasion. Our study has revealed that EGFR-AS1 contributes to the pathogenesis of NSCLC by inhibiting autophagic-lysosomal degradation via targeting the miR-524-5p/DRAM1 axis. This finding elucidated for the first time the role of EGFR-AS1 in lung cancer progression and the positive regulatory function of miR-524-5p in regulating DRAM1 protein and suppressing lung cancer progression. This novel mechanism provided a better insight into the pathogenesis of lung cancer and presented a better strategy for the treatment of lung cancer.

## 1. Introduction

Lung cancer is explicated as small cell lung cancer or nonsmall cell lung cancer (NSCLC), where NSCLC accounts for the majority of lung cancers and is a leading global cause of cancer-related deaths [1,2]. NSCLC is a heterogeneous disorder, with various subtypes representing different clinical indexes requiring different treatment strategies. Distinct clinical outcomes accompany these different histological subtypes, disclosing heterogeneity in disease aggressiveness and underlying prognostic alterations [3–5]. Indeed, complex cellular signaling and tumor microenvironment factors are associated with poor prognosis, imparting a distinctive biological basis to an individual's disorder [1]. The identification of oncogenic driver modulations has helped ameliorate the outcomes in lung cancer patients. However, most lung cancer patients do not have an actionable molecular abnormality [6,7]. Therefore, identifying new biomarkers and alternative treatments is of great necessity.

Long noncoding RNAs (lncRNAs) are RNA transcripts larger than 200 bp and encode no proteins [8–11]. lncRNA has specific patterns in healthy and tumor tissues. The most frequently expressed lncRNAs in lung cancers are long intergenic noncoding RNA, antisense RNA, and processed transcripts [12]. lncRNAs have emerged as novel cancer mediators [13–18], although most lncRNAs have yet to be discovered. lncRNAs seem to be engaged in cell proliferation, migration, differentiation, immune response, apoptosis, tumorigenesis, and angiogenesis [14,18–21]. Previously, epidermal growth factor receptor antisense RNA 1 (EGFR-AS1) was shown to promote cell cycle progression in hepatocellular carcinoma [22] and modulate squamous cell carcinoma treatment response [23]. In addition, EGFR-AS1 overexpression is associated with a poor prognosis and promotes chemotherapy resistance in NSCLC [24]. However, the underlying mechanism of EGFR-AS1 in NSCLC remains to be fully elucidated.

MicroRNAs (miRNAs) are endogenous, small noncoding RNAs that positively or negatively regulate gene expression [25]. Riveting evidence has elucidated the distinctly dysregulated miRNA expression in human cancers, including deletion or amplification of miRNA genes, dysregulated epigenetic changes, transcriptional control of miRNAs, and the flawed miRNA biogenesis mechanism [26]. miR-524-5p was reported to be essential in the pathogenesis of gliomas [27]. It has been recently found to be highly expressed in idiopathic pulmonary fibrosis (IPF) and can be utilized as a biomarker for the diagnosis and prognosis of IPF [28]. This finding indicated that miR-524-5p could potentially have a role in the pathogenesis of other lung-related disorders.

DNA damage-regulated autophagy modulator 1 (DRAM1) is known to induce autophagy and is downregulated in multiple human cancers [29]. In the presence of growth factors, DRAM1 regulates the activation of the IGF-1 receptor and inhibits the downstream PI3K-AKT-mTOR pathway, promoting autophagy activation and suppressing cell proliferation in various human cancers [30]. DRAM1 was reported to be decreased in NSCLC and was negatively correlated with EGFR levels. In addition, DRAM1 overexpression inhibited the proliferation, invasion, migration, and EMT of NSCLC cell lines harboring mutant EGFR *in vitro* and *in vivo* [31]. Nevertheless, the underlying mechanism of DRAM1 in the pathogenesis of NSCLC remains unclear.

The mechanism of EGFR-AS1 and miR-524-5p in lung cancer requires detailed analysis. On the other hand, the role of DRAM1 in lung cancer remains unclear. The present study identified EGFR-AS1/miR-524-5p/DRAM1 as a novel signaling pathway associated with lung cancer progression and comprehended the mechanism of miR-524-5p and DRAM1 in lung cancer pathogenesis.

## 2. Materials and Methods

### 2.1. Clinical Samples

Eighty pairs of cancerous and adjacent normal lung tissues were collected from patients diagnosed with lung cancer at Jinan Central Hospital Affiliated to Shandong University. The tissue specimens were collected postsurgical resection and promptly transferred and stored in liquid nitrogen. All patients had written informed consent. This study was approved by Jinan Central Hospital Affiliated to Shandong University.

### 2.2. Cell Lines and Cell Culture

The human nontumorigenic lung epithelial cell line BEAS-2B was purchased from the American Type Culture Collection (ATCC) and was cultured in BEBM complete medium supplemented with 10% FBS and incubated at 37°C and 5% CO_2_ atmosphere along with penicillin (100 U/ml) and streptomycin (100 mg/ml, HyClone, USA). The adenocarcinoma lung cancer cell lines HCC827 and NCI-H1650 were purchased from ATCC and were cultured in RPMI-1640 medium supplemented with 10% FBS and incubated at 37°C and 5% CO_2_ atmosphere along with penicillin (100 U/ml) and streptomycin (100 mg/ml, HyClone, USA) at 37°C with 5% CO_2_.

### 2.3. Cell Transfection

HCC827 and NCI-H1650 cells were transfected with constructed lentivirus vectors by RiboBio (Guangzhou, China) to knockdown the expression of miR-524-5p. According to the manufacturer's protocols, the cells were transfected accordingly using Lipofectamine 2000 transfection reagents (Invitrogen, Shanghai, China). The cell culture medium was replaced with a fresh medium after 24 h. In addition, cells were transfected with pcDNA3.1-si-EGFR-AS1 or miR-524-5p inhibitor (GenePharma, Shanghai, China). The empty vectors in this experiment were considered as the negative control. Lentiviruses (lv-shEGFR-AS1 and lv-oeEGFR-AS1) were purchased from Shanghai Heyuan Biotechnology and were transduced into HCC827 cells as previously described [32].

### 2.4. RT-qPCR

The total RNA from cultured cells was extracted using TRIzol reagent (Invitrogen, Carlsbad, CA, USA). Following the manufacturer's protocols, the cDNA was synthesized using M-MLV Reverse Transcriptase (Promega, Madison, WI, USA). miR-524-5p, EGFR-AS1, and DRAM1 mRNA levels were quantified. U6 was used as an internal control for miRNA, and GAPDH was used as an internal control for EGFR-AS1 and DRAM1. The relative expression levels of miR-524-5p, EGFR-AS1, and DRAM1 were calculated using the 2^-∆∆^CT method. The following primers were used in the experiments: *miR-524-5p*: forward: 5′-GTGCTCACTCCAGAGGGATG-3′, reverse: 5′-TATGGTTGTTCACGACTCCTTCAC-3′; *EGFR-AS1*: forward: 5′-CCATCACGTAGGCTTCCTGG-3′, reverse: 5′-GCATTCATGCGTCTTCACCTG-3′; and *DRAM1*: forward: 5′-CCACGAUGUAUACAAGAUA-3′, reverse: 5′-CCACGAAAUCAAUGGUGA-3′.

### 2.5. Cell Viability and Invasion Assays

The cell viability of HCC827 and NCI-H1650 cells was measured using the CCK-8 reagent (Sigma-Aldrich, St. Louis, MO, USA). Three thousand cells/well were seeded into the 96-well plate and then cultured for 24, 48, and 96 h. Cell viability was measured by detecting the absorbance of cultured cells using a microplate reader at 450 nm after the addition of CCK-8 for two hours.

The invasion ability of HCC827 and NCI-H1650 cells was assayed using the Matrigel-coated Transwell (Corning, MA, USA). Cells (2 × 10^4^) in 200 *μ*L of serum-free medium were added into the upper Transwell chambers. The bottom chamber was added with the complete medium. After incubation for 48 h in a humidified atmosphere (37 °C and 5% CO_2_), cells on the upper surface of the membrane were completely removed using a cotton swab. The remaining cells were fixed with methanol and stained for 20 min with 0.1% crystal violet. The stained cells were counted and assessed from five randomly selected fields under a microscope, and the data were presented from triplicate experiments.

### 2.6. Luciferase Report Assay

The binding of miR-524-5p to DRAM1 as well as their binding sequences were predicted from the miRDB database [33]. The wild-type EGFR-AS1 or DRAM1 3′untranslated region (3′UTR) sequences, which have binding sites with miR-524-5p, were inserted into the pmirGLO vector (Promega, Madison, WI, USA) to construct the WT-EGFR-AS1 or WT-DRAM1 vector. The mut-EGFR-AS1 or mut-DRAM1 vector was also constructed by inserting the mutated binding sequences into the pmirGLO vector. HCC827 and NCI-H1650 cells were cotransfected with luciferase reporter vectors and miR-524-5p mimic or negative vectors. Lastly, the relative luciferase activities were measured with a Dual-Luciferase Reporter Assay Kit (Promega, Madison, WI, USA) after 48 h of transfection.

### 2.7. Western Blotting

HCC827 and NCI-H1650 cells were harvested and lysed in the lysis buffer (Abcam, Cambridge, USA) containing PMSF and protease inhibitor (Roche, USA) for 15 min on ice. Afterward, the cell lysate was centrifuged at 12.000 g at 4°C for 10 min. The protein concentrations were calculated using the BCA Protein Assay kit (Sigma-Aldrich, MO, USA). An equal amount of protein was loaded and electrophoresed on a 10% sodium dodecyl sulfate-polyacrylamide gel electrophoresis, then transferred onto the polyvinylidene fluoride membrane (Millipore, Billerica, USA). Posttransfer, the membrane was blocked in 5% nonfat milk for one hour at room temperature. Then, the membranes were probed with primary antibodies against DRAM1 (1/2000; ab208160), SQSTM1 (1/10000; ab109012), Beclin-1 (1/2000; ab207612), LC3 (1/2000; ab192890), and GAPDH (1/2500; ab9485) overnight at 4°C, and incubated with the HRP conjugated secondary antibody IgG (1/10000; ab98624) for 1.5 h at room temperature. All antibodies were purchased from Abcam (Cambridge, USA).GAPDH polyclonal antibody was used as an internal control. The protein bands were visualized using an enhanced chemiluminescence visualization system (ECL Plus, Amersham Life Sciences).

### 2.8. In Vivo Xenograft Model

The present *in vivo* experiments were approved by the Animal Research Ethics Committee of the Jinan Central Hospital Affiliated to Shandong University. The 5-week-old female BALB/c nude mice were purchased from Charles River (Beijing, China) and subsequently randomly divided into the designated groups consisting of five mice in each group. HCC827 cells (2 × 10^7^) were injected subcutaneously into the right flank of each mouse. The tumor size was calculated and recorded once a week. Six weeks later, the tumors were extracted, photographed, and weighed.

### 2.9. Statistical Analysis

All data presented were analyzed for statistical significance using the Student's *t*-test and analysis of variance (ANOVA). Spearman's correlation analysis was used for expression correlation analysis in NSCLC specimens. Data in this study were obtained from three independent experiments and were presented as mean ± standard deviation (S.D). The software GraphPad Prism 8.0 (San Diego, CA) was used for all statistical analyses, and a *p* < 0.05 was considered significant.

## 3. Results

### 3.1. Silencing EGFR-AS1 Inhibited Lung Cancer Cell Viability and Invasion and Reduced Tumor Size *In Vivo*

**Figure 1:**
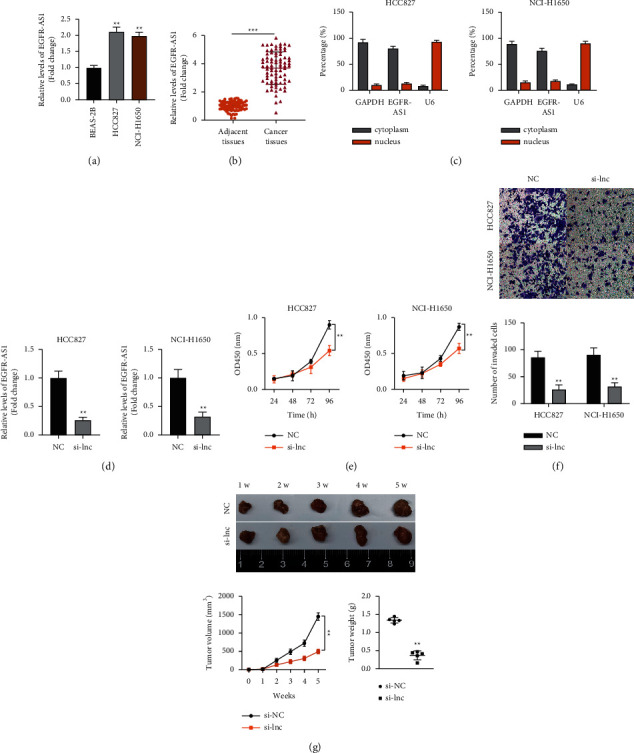
EGFR-AS1 is vital for NSCLC pathogenesis. (a) The relative expression level of EGFR-AS1 in normal BEAS-2B cells and NSCLC cell lines. One-way ANOVA was applied. (b) The expression levels of EGFR-AS1 in 80 samples of normal lung tissues and NSCLC tissues. The paired-student *t* test was applied. (c) Cytoplasmic and nuclear expression of EGFR-AS1 in NSCLC cells was detected by RT-qPCR. (d) The knockdown effect of si-EGFR-AS1 in NSCLC cells was assessed by RT-qPCR. The unpaired-student *t* test was applied. (e) The effect of EGFR-AS1 knockdown on NSCLC cells' viability. Two-way ANOVA was applied. (f) The effect of EGFR-AS1 knockdown on the NSCLC cells' invasion. The unpaired-student *t* test was applied. (g) The effect of EGFR-AS1 knockdown on the tumor weight and volume in nude mice bearing HCC827 cells. Two-way ANOVA and the unpaired-student *t* test were applied for difference comparison in the line chart and bar chart, respectively. The data are represented as mean ± S.D. ^*∗∗*^*p* < 0.01 and ^*∗∗∗*^*p* < 0.001.

We compared the expression level of EGFR-AS1 in normal lung cells and tumor cells. EGFR-AS1 is upregulated in HCC827 and NCI-H1650 lung cancer cells compared to normal BEAS-2B cells (Figure 1(a)). EGFR-AS1 was highly expressed in tumor tissues compared to healthy tissues (Figure 1(b)). EGFR-AS1 was more prominent in the cytoplasm in HCC827 and NCI-H1650 cells than in the nucleus (Figure 1(c)). We knocked down EGFR-AS1 expression in NSCLC cells, and it significantly inhibited its expression compared to the negative control group (Figure 1(d)). Next, we determined the effect of silencing EGFR-AS1 on cell viability and invasion. We found that silencing EGFR-AS1 significantly inhibited the viability and invasion of HCC827 and NCI-H1650 cells (Figures 1(e) and 1(f)). Simultaneously, silencing EGFR-AS1 reduced tumor size in nude mice bearing xenografts (Figure 1(g)).

### 3.2. EGFR-AS1 Expression Is Negatively Correlated with the Expression of miR-524-5p

**Figure 2:**
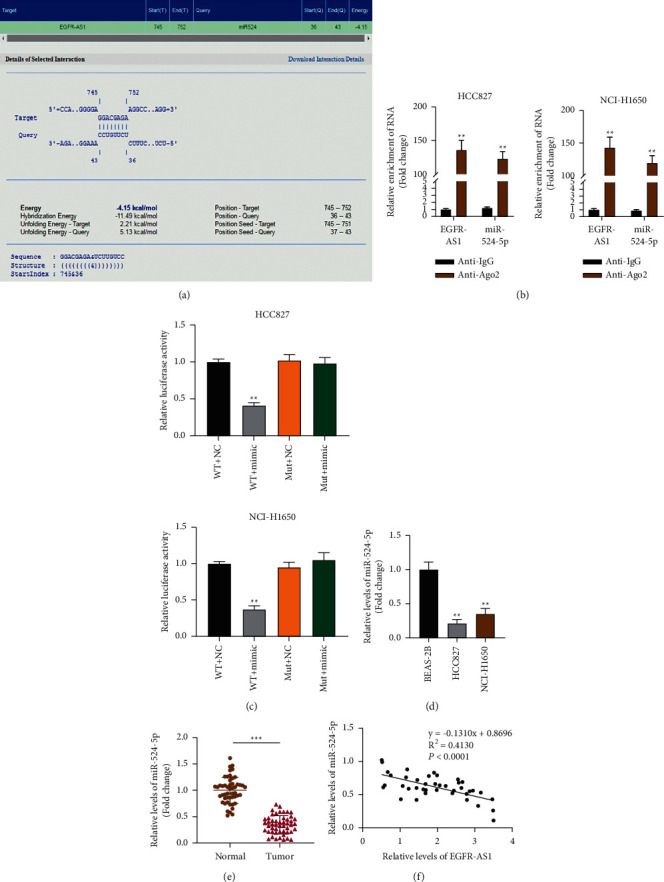
miR-524-5p is negatively correlated to EGFR-AS1. (a) In silico prediction of interaction between EGFR-AS1 and miR-524-5p. (b) Comparison of the different relative expressions of EGFR-AS1 and miR-524-5p enriched in Ago2 in NSCLC cells. The unpaired-student *t* test was applied. (c) The binding of miR-524-5p to EGFR-AS1 in NSCLC cells was assessed by luciferase reporter assay. The unpaired-student *t* test was applied. (d) The expression level of miR-524-5p in normal lung cells and NSCLC cells. One-way ANOVA was applied. (e) The expression level of miR-524-5p in 50 samples of normal lung tissues and NSCLC tissues. The paired-student *t* test was applied. (f) A negative correlation was found between miR-524-5p and EGFR-AS1 in NSCLC samples. Spearman's correlation analysis was conducted. The data are represented as mean ± S.D. ^*∗*^*p* < 0.05, ^*∗∗*^*p* < 0.01, and ^*∗∗∗*^*p* < 0.001.

We utilized in silico analysis to predict if there could be a potential relation/interaction between EGFR-AS1 and miR-524-5p. As shown in Figure 2(a), EGFR-AS1 and miR-524-5p have been shown to interact potentially. Comparing the enrichment levels of EGFR-AS1 and miR-524-5p, EGFR-AS1 is more enriched than miR-524-5p in HCC827 and NCI-H1650 cells (Figure 2(b)). To confirm the binding between EGFR-AS1 and miR-524-5p, we conducted a luciferase activity assay. Luciferase activity of wt-EGFR-AS1 was suppressed by miR-524-5p mimics, while that of mut-EGFR-AS1 showed no significant response (Figure 2(c)). On the other hand, the expression of miR-524-5p was found to be higher in healthy lung cells and tissues than in control cells and tissues (Figures 2(d) and 2(e)). Moreover, we found that EGFR-AS1 expression is negatively correlated with the expression of miR-524-5p (Figure 2(f)).

### 3.3. miR-524-5p Expression Suppressed Lung Cancer Cell Viability and Invasion

**Figure 3:**
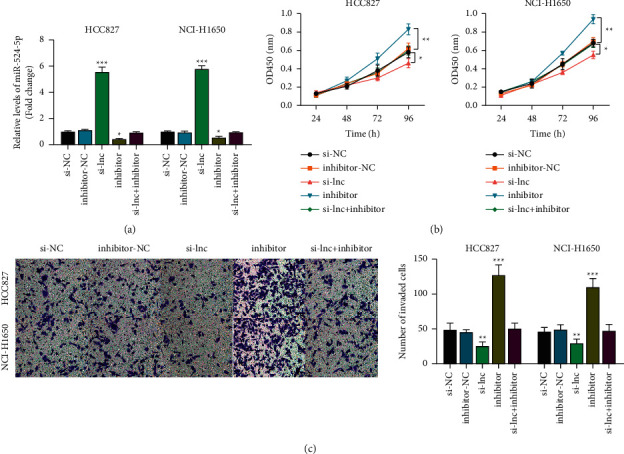
miR-524-5p inhibition promoted NSCLC cell progression. (a) The effect of si-EGFR-AS1 and miR-524-5p inhibitors on the miR-524-5p expression level. One-way ANOVA was applied. (b) The CCK-8 assay determined the effect of inhibiting miR-524-5p and EGFR-AS1 on NSCLC cell viability. Two-way ANOVA was applied. (c) The effect of inhibiting miR-524-5p and EGFR-AS1 on NSCLC cell invasion. One-way ANOVA was applied. The data are represented as mean ± S.D. ^*∗*^*p* < 0.05, ^*∗∗*^*p* < 0.01, and ^*∗∗∗*^*p* < 0.001.

We analyzed the role of miR-524-5p in the progression of lung cancer cells. We found that silencing EGFR-AS1 promoted the expression of miR-524-5p while miR-524-5p inhibited its expression (Figure 3(a)). Inhibiting miR-524-5p increased lung cancer cell viability, while silencing EGFR-AS1 decreased it compared to the negative control group. These effects were rescued by the combination of si-EGFR-AS1 and miR-524-5p inhibitor (Figure 3(b)). We determined the effect of inhibiting miR-524-5p on cell invasion. Our results indicated that compared to the negative control group, inhibiting miR-524-5p significantly increased lung cancer cell invasion while silencing EGFR-AS1 significantly inhibited cell invasion. These effects were rescued by si-EGFR-AS1+miR-524-5p inhibitor (Figure 3(c)). These results indicated the correlation between EGFR-AS1 and miR-524-5p.

### 3.4. miR-524-5p Negatively Correlated with the Expression of DRAM1 in Lung Cancer Cells

**Figure 4:**
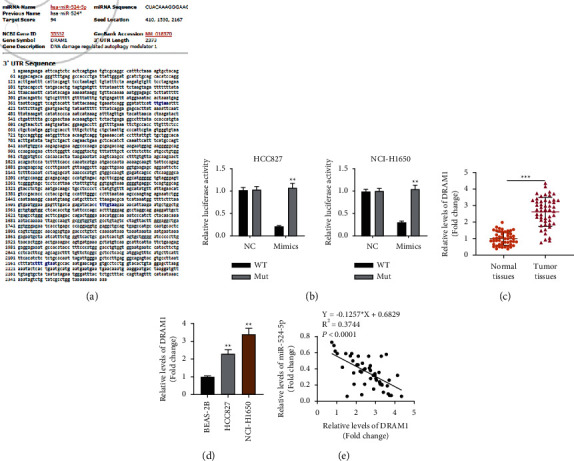
The expression of miR-524-5p is negatively correlated with DRAM1 expression. (a) In silico prediction of the potential targets of miR-524-5p. (b) The binding of miR-524-5p on DRAM1 was revealed by luciferase reporter assay. The unpaired-student *t* test was applied. (c) The expression level of DRAM1 in 50 samples of normal lung tissues and NSCLC tissues. The paired-student *t* test was applied. (d) The expression level of DRAM1 in normal lung cells and NSCLC cells. One-way ANOVA was applied. (e) There was a negative correlation between the expression of DRAM1 and miR-524-5p. Spearman's correlation analysis was conducted. The data are represented as mean ± S.D. ^*∗*^*p* < 0.05, ^*∗∗*^*p* < 0.01, and ^*∗∗∗*^*p* < 0.001.

To identify the potential target of miR-524-5p, we utilized *in silico* analysis to recognize the potential targets of miR-524-5p (Figure 4(a)). miR-524-5p reduced luciferase activity of DRAM1-wt and had no significant effects on that containing DRAM1-mut (Figure 4(b)). Knowing that DRAM1 is downregulated in lung cancer cells, we confirmed these observations *in vitro* and *in vivo*; we found that DRAM1 is upregulated in NSCLC tissues compared with adjacent nontumor tissues. DRAM1 was upregulated in the HCC827 and NCI-H1650 cell lines than the control BEAS-2B cells (Figures 4(c) and 4(d)). Finally, we found that the expression of miR-524-5p was negatively correlated with DRAM1 expression (Figure 4(e)). This result indicated a possibility that miR-524-5p could have a regulatory role on DRAM1.

### 3.5. The miR-524-5p/DRAM1 Axis Is Vital for Lung Cancer Cell Biological Processes

**Figure 5:**
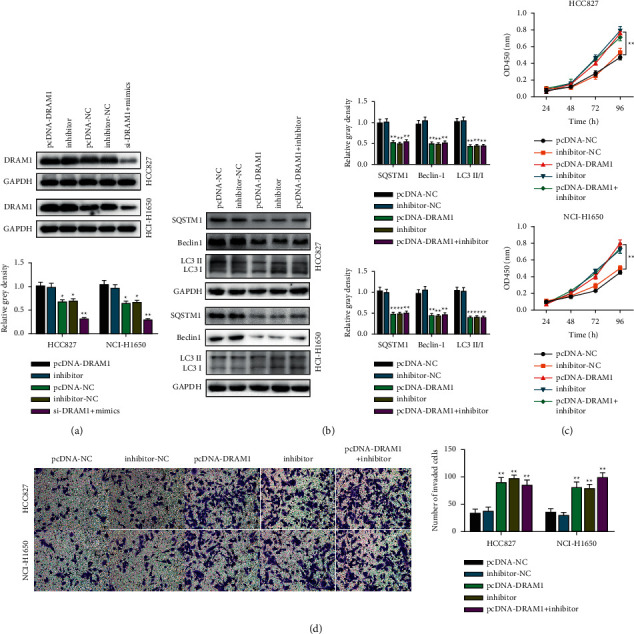
The miR-524-5p/DRAM1 axis is vital for lung cancer cell biological processes. (a) Western blotting analysis was utilized to determine the effect of miR-524-5p inhibition on DRAM1 protein expression in NSCLC cells. One-way ANOVA was applied. (b) Western blot analysis was utilized to determine the effect of miR-524-5p inhibition on autophagy-related protein expression in NSCLC cells. One-way ANOVA was applied. (c) The CCK-8 assay was used to determine the effect of miR-524-5p inhibition and DRAM1 knockdown on NSCLC cell viability. Two-way ANOVA was applied. (d) The effect of inhibiting miR-524-5p and DRAM1 on NSCLC cell invasion. One-way ANOVA was applied. The data are represented as mean ± S.D. ^*∗∗*^*p* < 0.01.

We further investigated the role of DRAM1 in lung cancer cell biological processes. Western blot analysis showed that both pcDNA-DRAM1 and miR-524-5p inhibitors increased DRAM1 protein expression (Figure 5(a)). Concerning the role of DRAM1 in autophagy, we examined the autophagic signaling pathway and found that the inhibition of miR-524-5p and overexpression of DRAM1 suppressed SQSTM1, Beclin-1, and LC3 protein expression compared to the negative control groups (Figure 5(b)). CCK-8 and transwell assays showed that overexpression of DRAM1 and inhibition of miR-524-5p significantly promoted lung cancer cell viability and invasion compared to the negative control groups (Figures 5(c) and 5(d)).

## 4. Discussion

Globally, the lung cancer mortality rate is very high. NSCLC represents most of the disease despite improving NSCLC management [34,35]. This burden turned scientists' attention toward lncRNAs as a potential therapeutic target in the treatment of NSCLC, which play essential roles in gene expression and signaling pathways [36–38]. Recently, lncRNAs have been dysregulated in different cancer types, resulting in aberrant cell functions. They could act as tumor suppressors and oncogenes in different cancer types [12,39]. EGFR-AS1 is engaged in the progression of lung cancer [24]. EGFR-AS1 has been reported to be upregulated in lung cancer and has been significantly correlated with the poor survival of lung cancer patients [24]. After knockdown, the EGFR-AS1 gene expression profiling gives more unbiased information concerning pathways at the transcriptional levels. The knockdown of EGFR-AS1 has been shown to suppress lung cancer cell migration, invasion, and proliferation [24]. Our experimental results confirmed the previously reported findings. We have confirmed that the knockdown of EGFR-AS1 inhibited cell viability in HCC827 and NCI-H1650 cell lines and reduced tumor size *in vivo* in nude mice models bearing the HCC827 cells.

miRNAs can sustain proliferative signaling, evade growth suppressors, avoid immune destruction and tumors, promote inflammation, resist cell death mechanisms, deregulate cell energetics, activate invasion and metastasis, and induce angiogenesis [40]. Increased attention has been drawn toward miR-524 in human cancers. Its downregulation was reported to suppress angiogenesis in colon cancer and promote cell proliferation in osteosarcoma [41,42]. It could be used as a biomarker for the early diagnosis of idiopathic pulmonary fibrosis [28]. Moreover, the overexpression of miR-524-5p is associated with a poor prognosis of patients with idiopathic pulmonary fibrosis [28]. Considering the previously reported findings [24,28,31], we examined the role of miR-524-5p in HCC827 and NCI-H1650 cell lines and found that the enrichment of miR-524-5p was significantly lower than EGFR-AS1, and that they were inversely expressed in HCC827 and NCI-H1650 cell lines. Subsequently, the inhibition of miR-524-5p promoted the viability and invasion of HCC827 and NCI-H1650 cell lines. These results indicated that the lncRNA EGFR-AS1 could have a regulatory role over miR-524-5p.

Emerging evidence suggested that DRAM1 is engaged in the biological functions of cancer cells [30,43]. DRAM1 has been found to play a tumor suppressor role in NSCLC [31]. However, DRAM1 expression and clinical significance in lung cancer have not been elucidated. Considering the vital role of miR-524-5p in lung cancer prognosis [28], we explored its potential in regulating DRAM1 expression. We have found that miR-524-5p decreased DRAM1 expression. There was a negative expression correlation between miR-524-5p and DRAM1. Inhibiting miR-524-5p or overexpressing DRAM1 decreased autophagy-related proteins and increased viability and invasion in NSCLC cells.

Our results suggested that EGFR-AS1 could regulate NSCLC progression by the DRAM1/miR-524-5p axis. For the first time, we reported DRAM1 as a potential target of miRNA-524-5p and elucidated its role in lung cancer. The present study has some limitations. We shall investigate the role of EGFR-AS1 concerning the miR-524-5p/DRAM1 axis *in vivo* and provide a detailed analysis of the underlying mechanism. We also need to validate our results regarding cell death mechanisms.

## 5. Conclusion

We spotlighted the vital role of EGFR-AS1 in NSCLC progression and the role of miR-524-5p in regulating DRAM1. For the first time, our results have practically elucidated that EGFR-AS1 contributes to the progression of NSCLC via inhibiting the miR-524-5p/DRAM1 axis. Our findings set forth a novel mechanism of the progression of NSCLC, providing a newly identified target for lung cancer treatment.

## Data Availability

Data supporting this research article are available from the corresponding author on reasonable request.
